# Assessing the Efficacy and Long-Term Outcomes of Surgical Intervention Versus Radiotherapy: A Comprehensive Systematic Review and Meta-Analysis of Prostate Cancer Treatment Modalities

**DOI:** 10.7759/cureus.58842

**Published:** 2024-04-23

**Authors:** Tauqir Aslam Waraich, Syed Yousaf Khalid, Usama Muhammad Kathia, Azfar Ali, Saleem Shahzad Shumas Qamar, Ammar Yousuf, Rana Muhammad Umair Saleem

**Affiliations:** 1 Department of Urology, Letterkenny University Hospital, Letterkenny, IRL; 2 Department of General Surgery, Letterkenny University Hospital, Letterkenny, IRL; 3 Department of Cardiothoracic Surgery, St. James's Hospital, Dublin, IRL; 4 Department of Urology and Kidney Transplantation, Lahore General Hospital, Lahore, PAK; 5 Department of Urology, Pakistan Kidney and Liver Institute and Research Center, Lahore, PAK

**Keywords:** treatment outcomes, brachytherapy, external beam radiation therapy, radical prostatectomy, advanced prostate cancer

## Abstract

There is controversy regarding the most effective primary treatment of choice for prostate cancer (PCa) in terms of patient outcomes, such as surgery or radiotherapy (RT). This study evaluated the comparative efficacy and long-term outcomes of radical prostatectomy (RP) and RT for PCa treatment. A thorough literature review of relevant databases was conducted, focusing on academic and clinical studies published from 2019 onwards. The inclusion criteria included randomized controlled trials (RCTs) and other observational studies comparing survival outcomes in patients treated with surgery and RT. We followed the Preferred Reporting Items for Systematic Review and Meta-Analysis (PRISMA) guidelines to provide an overview of the data. We selected 19 studies based on the inclusion criteria. Of the total 19 studies, 12 advocated RP as the preferred treatment to improve survival outcomes in patients with PCa. The results of our synthesis showed that prostate cancer-specific mortality (PCSM) was lower in patients treated with RT. The total effect size for the analysis was calculated as Z=1.19 (p-value=0.23). The heterogeneity in the studies was as follows: Tau2=0.09, Chi2=20.25, df=4, I2=80%. Moreover, overall survival (OS) was shown to be higher in patients who underwent prostatectomy. The combined effect for the analysis was found to be: HR=0.97 (0.93, 1.01). The total effect was calculated as Z=1.33 (p-value= 0.18). The heterogeneity was found to be Tau2=0.00, Chi2=1.33, df=2, and I2=0%. However, overall mortality (OM) was shown to be independent of the treatment modality. RT is the preferred strategy for PCa treatment, as it balances efficacy and long-term outcomes. Clinical decision-making should consider individual patient characteristics and future research should delve into specific subpopulations and long-term outcomes to further refine the treatment guidelines.

## Introduction and background

Prostate cancer (PCa) is the second most frequently diagnosed cancer and the fifth leading cause of cancer-related deaths among men globally. In 112 nations, PCa is the most common malignancy to be diagnosed; in 48 countries, it is the primary cause of cancer-related deaths [1]. It is important to note that as the population ages and the economy grows, there is likely to be a rise in the prevalence of PCa [2]. PCa metastasizes to various organs, with a greater proclivity for the bone [3]. Up to 10% of patients with PCa already have bone metastases identified at the time of the initial diagnosis, despite the early discovery of the main tumor. Radiation therapy (RT), chemotherapy, hormone therapy, cryosurgery, and other techniques are some of the current medical treatment modalities. These methods work reasonably well when used alone or in combination with other methods. However, these methods have several undesirable side effects. Constant research is being conducted to determine the best course of action or means of minimizing adverse impacts [4]. A comprehensive randomized controlled study (RCT) comparing radical prostatectomy (RP), external beam radiation therapy (EBRT), and active monitoring for the treatment of localized PCa was reported in 2016 [5]. The updated data on the oncological results of RP versus EBRT for localized PCa were compiled in this analysis and review.

Rationale

Determining the most efficacious treatment modality for PCa can help provide early symptomatic relief to patients and secure patient prognosis, ensuring improved clinical outcomes. Limited records of updated evidence are available on the efficacy of surgical and radiation intervention modalities on patient survival and long-term outcomes, such as recurrence, metastasis, biochemical progression, and urinary and sexual function. It is important to determine the different efficacies of these treatment modalities through evidence-based studies to be able to create clinically oriented guidelines that medical practitioners and surgeons can use for the effective development of treatment strategies that cater to the specific needs and requirements of PCa patients. Understanding the comparative effectiveness of prostatectomy and RT is crucial for tailoring PCa therapy to individual patient needs, optimizing adherence, and improving clinical outcomes.

Objectives

The current investigation aimed to evaluate the efficacy of surgery and RT in the treatment of PCa through outcomes such as cancer-specific mortality (CSM) and all-cause/overall mortality (ACM or OM); compare the different effects of these treatments on long-term patient outcomes; and provide insight for future research on the topic and help create guidelines for medical practitioners concerning PCa treatment.

Definitions

PCa is characterized by the uncontrolled growth of cells in the prostate gland [6]. Prostatectomy, a surgical procedure, involves either partial or complete removal of the prostate [7]. RT, on the other hand, employs ionizing radiation to eliminate cancer cells [8]. Efficacy in the context of PCa treatment refers to the ability of RP or RT to effectively treat the disease and prevent mortality. Long-term outcomes encompass a spectrum of changes that occur several years after intervention, including patient satisfaction, toxicity, biochemical recurrence, metastasis, clinical progression, urinary and sexual activity, and overall quality of life. CSM reflects the number of deaths in a specific population where cancer is the underlying cause within a given time frame [9], while OM represents the risk of death from any cause, including disease, complications, accidents, or exposures [10]. Overall survival (OS) is defined as the time from treatment initiation to death, irrespective of disease recurrence [11].

## Review

Methods

Eligibility Criteria

We set the eligibility criteria according to the ‘Population, Intervention, Comparison, Outcome, and Study Design (PICOS)’ scheme, as recommended by PRISMA guidelines.

The inclusion criteria were as follows: literature published from 2019 to 2024; adult males with a confirmed diagnosis of PCa; studies investigating the efficacy and long-term outcomes of various PCa treatment modalities; studies comparing prostate surgery with RT; and studies reporting efficacy in survival and long-term Quality of Life (QoL) outcomes.

The exclusion criteria were as follows: studies published before 2019; non-observational studies and other review studies were not selected; studies with a target population of diagnoses other than PCa; and studies that included a young pediatric population.

Information Sources

Several digital databases have been used to retrieve relevant studies. These include ClinicalTrials.gov, PubMed, Google Scholar, ScienceDirect, Medline, Embase, and the Cochrane Library. There are also independent journals and other sources. Other than databases, the literature was sourced from publications like "JAMA Oncology," "Radiation Oncology Journal," "BMJ," "Elsevier," "The New England Journal of Medicine," and others.

Search Strategy

The search strategy was established based on the PICOS scheme (discussed later) and was aimed at retrieving only the most relevant data from the digital databases. Using the current search strategy, 19 studies (out of a total sample of n=369) were eligible. We developed the PubMed search string and covered the terms: ("Prostatic Neoplasms" OR "prostate cancer") AND ("Therapeutics" OR "Radiotherapy" OR "Prostatectomy" OR "Treatment Outcome" OR "Surgery" OR "Radiotherapy"), filters: abstract, free full text, full-text clinical study, randomized controlled trials in the last five years, humans, English.

Selection Process

Four researchers reviewed the peer-reviewed journals and publications that met the inclusion criteria. Peer-reviewed journals with a high impact factor were investigated after careful selection of the literature to lower the possibility of publication bias. For primary and secondary literature screening, all selected studies were uploaded to Rayyan.ai, a screening program [12]. Four researchers collaborated to "include" or "exclude" appropriate studies based on the inclusion and exclusion criteria. Nineteen studies (n=369) were considered for the final review and analysis. Research that failed the eligibility requirements for screening was classified as "dispute" or "exclusion." We assembled a team of four researchers for study selection to act as tiebreakers for disputed studies. Studies that had a different population, had a design and methodology that were not appropriate for inclusion, calculated incorrect outcomes, or had a high-risk of bias were all excluded. Occasionally, we observed a combined effect of several exclusionary factors.

Data Items

**Figure 1:**
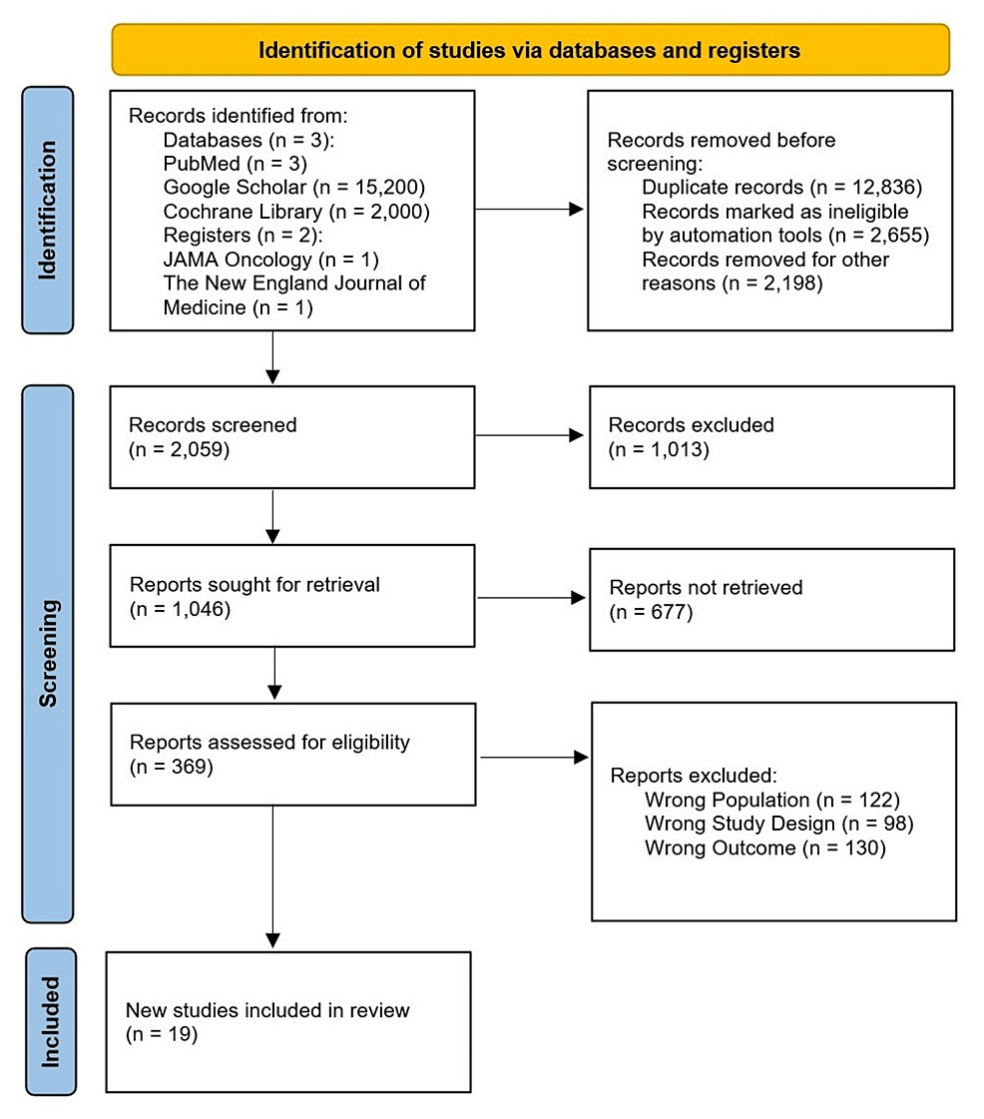
PRISMA flow chart for selected studies PRISMA: Preferred Reporting Items for Systematic Review and Meta-Analyses

Following the completion of the secondary screening protocol, the total sample size (n=19) for the chosen literature was evaluated. For the chosen studies from journals and other independent resources (if the reports were available), we created a PRISMA flow diagram using the Preferred Reporting Items for Systematic Review and Meta-Analysis (PRISMA) standards [13] (Figure 1).

To reduce bias in the analysis, the following measures were taken: choosing high-quality research; requiring peer reviewers to disclose conflicts of interest; and substituting meta-analyses for regular review articles. Systematic and narrative reviews were excluded to maintain the standards of the study. Following the stages of removing publication bias proposed by Chalmers et al. (1990), these guidelines identified and eliminated bias from the study protocol [14].

Assessment of Research Quality

Systematic review: Study bias was examined in all primary studies selected for the assessment of quality. The demographics of the population, the studies' intervention features, and their outcome domains were manually reviewed. All studies selected for meta-analysis underwent quality assessment using the Critical Appraisal Skill Program (CASP) tool. The quality assessment included three broad question categories: were the study results validated, what are the results, and are the results of this study locally applicable? Eleven questions for quality assessment were answered with careful consideration of the study designs and relevant outcomes. The responses to the questions were "yes," "no," and "i can't tell.” If the first question is answered in the affirmative, it makes logical sense to move on to other inquiries. The questions overlapped with each other in certain ways. The description of the answers and the researchers' remarks have also been mentioned in the assessment table (see Results section).

Meta-analysis: To evaluate ‘bias’ in the studies that were chosen, we looked for digital and online tools. Except for RCTs, every study was evaluated using an online tool through CASP to produce a quality assessment table for every study, which was a part of the meta-analysis. Additionally, every primary study, that is, all RCTs that qualified for analysis was chosen on its own using the Cochrane criteria for risk of bias (ROB). The domains with potential for bias were [15]: random sequence generation; allocation concealment; participant and personnel blinding; outcome assessment blinding; incomplete outcome data (attrition bias); selective reporting (reporting bias); and other biases. For the statistical meta-analysis, data that were given as generic inverse variance were obtained from 13 of the 19 primary studies. For the meta-analysis, we used Review Manager (RevMan version 5.4) to create a "forest plot." Rev-Man (version 3.5.1) was used to conduct a meta-analysis of ten primary studies (study design=cohort study). For the analytical tool, four researchers gathered comparable and poolable data [16]. Some data were available as generic inverse variances and the rest as dichotomous variables. The results section of our study contains meta-analysis data.

Results

Study Characteristics

We manually selected 19 studies for the final analysis. Of these, 18 were cohorts, and one was a randomized control trial. Twelve of these studies used Cox regression methods for the statistical analysis of prostate cancer-specific mortality (PCSM) or overall mortality (OM), and seven used the Kaplan-Meier method. Two studies used other propensity-scoring methods. Age, prostate-specific antigen (PSA), clinical T stage, biopsy Gleason Score (GS), race, and diagnosis year were utilized as factors in multivariable competing risk regression models in the majority of studies, which assessed the impact of various therapies on CSM and OM. One study used the Fine and Gray proportional hazard regression model. The sample size of the populations in each study ranged from N=156 to 137,741 (131,345 + 6396). The follow-up period ranged from three to 15 years. The results of the systematic review revealed a total of 12 out of 19 (63%) studies advocating the effectiveness of RP for the treatment of PCa. In contrast, one out of 19 (5.3%) studies advocated RT or brachytherapy (BT) with or without androgen deprivation therapy (ADT) to be effective in PCa treatment. Six out of the 19 (31.6%) studies also concluded that there was “no statistical difference” in either treatment modality on the outcomes of patients with PCa. A comparative analysis of prostatectomy versus RT showed no association between these interventions and patient outcomes in the majority of the available literature. The synthesis for the systematic review is presented in Table 1.

**Table 1:** Summary table of all included studies ACM: all cause mortality; ADT: androgen deprivation therapy; BCR: biochemical recurrence; BT: brachytherapy; BT-RT: brachytherapy-radiotherapy; CSM: cancer-specific mortality; DMFS: distant metastasis-free survival; DRE: digital rectal examination; EBBT: external beam brachytherapy; EBRT: external beam radiation therapy; GS: Gleason score; IMRT: intensity-modulated radiation therapy; IPTW: inverse probability of treatment weighting; JHU: John Hopkins University; LDR-BT: low dose rate brachytherapy; MDPCC: multidisciplinary prostate cancer clinic; MRI: magnetic resonance imaging; NCCN: National Comprehensive Cancer Network; OCM: other-cause mortality; OM: overall mortality; OS: overall survival; PCa: prostate cancer; PCSM: prostate cancer-specific mortality; PCSS: prostate cancer-specific survival; PLCO: Prostate, Lung, Colorectal, and Ovarian Cancer Screening Trial; protect: prostate testing for cancer and treatment; PSA: prostate specific antigen; RP: radical prostatectomy; RT: radiation therapy; SEER: Surveillance, Epidemiology, and End Results; SEED-BT: SEED brachytherapy; SPIRIT: Standard Protocol Items Recommendations for Interventional Trials

Sr. #	Study ID	Location	Study Design	Participants	Intervention	Main Findings
1	Benedikt Hoeh, 2021 [17]	USA	Retrospective cohort study	African American patients ≥18 years old within the SEER database (2010-2016); with adenocarcinoma of the prostate diagnosed at biopsy.	Cumulative incidence plots and competing risk regression models were made for CSM before and after 1:1 propensity score matching between RP and EBRT.	In JHU very high-risk African American patients, RP may hold a CSM advantage over EBRT.
2	Ugo Giovanni Falagario, 2023 [18]	Stockholm, Sweden	Retrospective cohort study	Male patients in the Stockholm PSA and biopsy register who underwent RT or RP with curative intent (cT1-3, cM0) and had at least one PSA after treatment.	To assess the effect of having a low or high-risk BCR and not having a BCR on PCSM, a competing-risk regression was fitted (with the competing risk of other-cause mortality). Age at diagnosis, D'Amico risk groups, treatment year, Charlson comorbidity index (CCI), timing of salvage treatment, and BCR risk groups were eventually incorporated into the model.	While the probability of BCR is significantly higher after RP, the risk of dying from PCa was higher after RT.
3	Nikhil T. Sebastian, 2019 [19]	USA	Retrospective cohort study	Patients up to the age of 70, with a Charlson/Deyo comorbidity score of 0 to 1, diagnosed with clinical T3N0M0 prostate adenocarcinoma according to NCCN criteria for unfavorable intermediate-disease: PSA 10 to 20 ng/ml, GS of 7, T-stage T2b to T2c, primary Gleason pattern 4, number of positive biopsy cores ≥50%, and/or presence of >1 intermediate-risk factor.	Cox regression was utilized for multivariable analysis, and Analysis of Deviance was employed to evaluate relationships between multilevel variables (n levels > 2). Based on factors such as age, race, year of diagnosis, T-stage, GS, PSA, type of treatment facility, proximity to hospital, population density, and education, cases were matched (pairwise) 1:1.	There was no statistically significant difference in OS between RP and EBRT+BT. RP was associated with higher survival when compared to EBRT.
4	Kirsti Aas, 2021 [20]	Norway	Retrospective cohort study	Patients diagnosed with high-risk PCa during 2006–2015, through the Cancer Registry of Norway, treated with RP 12 mo or RT 15 mo after diagnosis, and classified with high-risk disease according to the European Association of Urology (PSA >20 ng/ml or GS 8–10 or clinical tumor category 2c).	PCSM and OM were estimated using competing risk and Kaplan-Meier techniques, respectively. Hazard ratios (HRs) for PCSM and OM were assessed using multivariable Cox regression models.	Compared with RP, EBRT 70–<74 Gy was associated with increased and BT-RT with decreased 10-yr PCSM. Patients treated with EBRT 70–78 Gy had higher adjusted 10-yr OM than those treated with RP. In men with high-risk PCa, treatment with EBRT <74 Gy was associated with increased adjusted 10-yr PCSM and OM, and BT-RT with decreased 10-yr PCSM, compared with RP.
5	Derya Tilki, 2018 [21]	USA, Germany	Retrospective cohort study	639 men with clinical T1-4, N0M0 biopsy GS 9-10 PCa.	After fixing for treatment propensity score (PS), salvage therapy, and year of treatment, it was determined if PCSM risk and ACM risk, respectively, had a significant correlation with treatment using univariate and multivariable regression applying methods by Cox15 and Fine and Gray16.	There was no significant difference in the risk of PCSM and ACM when comparing men who underwent MaxRP vs MaxRT.
6	Francesco Chierigo & Marco Borghesi, 2022 [22]	USA	Retrospective cohort study	Patients ≥18 years old within SEER database 2004-2016 with histologically confirmed nonmetastatic, cN1 adenocarcinoma of the prostate, diagnosed at biopsy.	Following 1:1 propensity score matching (PSM), the impact of RP versus RT on OM was examined using Kaplan-Meier plots and Cox regression models; CSM and OCM between RP and RT patients were examined using cumulative incidence plots and competing-risks regression (CRR) models. Following the IPTW, all analyses were performed again.	RP may hold a CSM advantage over RT in cN1 PCa patients.
7	Omar Abdel-Rahman, 2019 [23]	USA	Retrospective cohort study	Patients who were diagnosed with clinically localized PCa within the PLCO trial and subsequently received treatment with prostatectomy or RT (with or without hormonal treatment).	The variables influencing OS and PCSS were identified using univariate and multivariate Cox regression analyses. In the multivariate analysis, factors that had a P < .05 in the univariate analysis were included.	Prostatectomy was associated with better OS compared with RT. Likewise, prostatectomy was associated with better PCSS compared with RT.
8	Fei Wang, 2021 [24]	USA	Retrospective cohort study	Patients with PCa from within the SEER and PLCO trials. N=131,345 (74,663 treated by RP and 56,682 by EBRT).	The 95% confidence intervals (CIs) and hazard ratios (HRs) were computed using Cox regression.	In both cohorts, patients who received RT exhibited a worse prognostic outcome than those who underwent RP.
9	Yu-Cheng Lu, 2022 [25]	Taiwan	Retrospective cohort study	Patients with clinical T stage 3/4 PCa defined by MRI. N=309 (111 treated by RP and 198 by EBRT).	BCR, local recurrence, metastasis, and OS were among the oncologic outcomes for which associations between clinical factors and Cox proportional hazards models were examined using both univariate and multivariate models.	Among locally advanced PCa patients, treatment with RP had a higher risk of BCR compared to the RT group. Treatment with RT plus ADT significantly decreased the risk of biochemical failure, but there was no significant difference in local recurrence, distant metastasis, and OS.
10	Seok-Joo Chun, 2021 [26]	Korea	Retrospective cohort study	Patients with PCa diagnosed via biopsy, with at least one high-risk feature according to the NCCN guidelines (initial PSA ≥20 ng/mL, GS ≥8, or clinical T stage ≥3a), and having undergone either RP or EBRT.	Prostatectomy included both open retropubic prostatectomy and robotic surgery. Pelvic lymphadenectomy was performed in half of the instances. IMRT was administered to the majority of patients. The patients underwent traditional fractionated RT before 2015, with a median dose of 81 Gy administered over 45 fractions. Following the release of hypofractionation randomized controlled trials, 70 Gy in 28 fractions became the standard.	The estimated 10-year PCSS was 97.0% in the RP and 95.9% in the EBRT. No significant difference was seen in the DMFS, whereas there was a trend in favor of RP over EBRT in OS. No significant difference in DMFS, PCSS, or OS was found in the propensity score matching analysis.
11	Freddie .C. Hamdy, 2023 [27]	UK	Randomized control trial	Patients taken from within the PCa cohort of the ProtecT trial. N=1643 (553 treated by RP and 545 by EBRT).	On an intention-to-treat basis, Cox proportional-hazards regression was employed to evaluate PCSM at 15 years in the three groups after adjusting for trial center, patient age, GS, and baseline PSA (log-transformed).	After 15 years of follow-up, PCSM was low regardless of the treatment assigned. Death from PCa occurred in 45 men (2.7%): 17 (3.1%) in the active-monitoring group, 12 (2.2%) in the prostatectomy group, and 16 (2.9%) in the RT group. Death from any cause occurred in 356 men (21.7%), with similar numbers in all three groups. Metastases developed in 51 men (9.4%) in the active-monitoring group, in 26 (4.7%) in the prostatectomy group, and in 27 (5.0%) in the RT group. Clinical progression occurred in 141 men (25.9%), 58 (10.5%), and 60 (11.0%), respectively.
12	N. Sanmamed, 2023 [28]	Europe	Retrospective cohort study	Patients from the SPIRIT trial who had favorable risk PCa (T1-T2a, PSA ≤10 ng/ml, Gleason score ≤6, were candidates for both RP and LDR-BT; had prostate volume <60 cm3, Eastern Cooperative Oncology Group performance status 0–2 and with no major medical comorbidities.	Using an ultrasound-guided approach, LDR-BT was performed on the prostate by implanting iodine-125 interstitial seed at a dosage of 145 Gy. Pre-plan dosimetry was designed to give 120-125% of the recommended dosage to 90% of the prostate (D90), and >100% of the prescribed dose to 99% of the prostate (V100 ≥ 99%). V200 was restricted to 12-20% of the recommended dosage, and V150 to 58-62%. Less than 150% of the recommended dosage could be administered to the urethra at any one time. After one month, all patients had post-implant computed tomography/magnetic resonance dosimetry.	The cumulative incidence function of biochemical failure was 0%, 1.1%, and 2.4% at 5, 10, and 15 years, respectively, in the LDR-BT arm versus 8.5%, 15.8%, and 15.8% in the RP arm. At 15 years, OS was higher in patients treated with RP compared with those treated with LDR-BT; however, no statistical difference was found in PCSS.
13	Sebastian Berg, 2018 [29]	USA	Retrospective cohort study	Young (65 yr) and healthy men (Charlson Comorbidity Index=0) with high-risk localized PCa in the National Cancer Database, diagnosed from 2004 to 2009.	Kaplan-Meier curves with IPTW-adjusted values were utilized to compare OS between the two groups. Additionally, a Cox regression model adjusted for IPTW was fitted to compare the groups' risks of OM.	EBRT + BT was associated with a higher risk of ACM compared with RP. RP showed statistically significant OS benefit compared with EBRT + BT.
14	Ahmed Emam, 2021 [30]	USA	Retrospective cohort study	Cases with newly diagnosed NCCN high or very high risk localized PCa, having gotten primary treatment at Roswell Park Comprehensive Cancer Center (RPCCC) since 2006, with more than 2 years follow‐up.	Kaplan-Meier techniques were used to summarize the survival results, and log-rank tests were used to compare them. Cox regression models were employed to assess the relationships between BF and clinical features in every group.	RP had higher rates of biochemical failure and adjuvant or salvage treatment versus EBRT in high-risk localized PCa. MFS trended toward benefit after EBRT, but CSS and OS remained high in both groups.
15	Xiao-Xiao Guo, 2021 [31]	China	Retrospective cohort study	Localized PCa patients aged ≥70 years identified from the 18th tumor registry of SEER database, who underwent RP, EBRT, BT, or EBBT between 2004 and 2016.	The effects of various treatments on CSM and OCM were assessed using multivariable competing hazard regression analyses, with factors including age, PSA, clinical T stage, biopsy GS, race, and diagnosis year. Using the competing risks approach, cumulative incidence smoothed plots were created for CSM and OCM, and 10-year CSM and OCM rates were computed.	In low- to intermediate-risk patients, there was no significant difference in CSM risk between RP and the other three RT modalities. In high-risk patients, EBRT was associated with a higher CSM than RP, whereas there was no significant difference between RP and BT, or RP and EBBT. Regarding OCM, the risk was generally lower in RP than in the other three RT modalities. In patients with high-risk diseases, RP is more beneficial than EBRT.
16	Sophie Knipper, 2019 [32]	USA	Population-based cohort	Clinically localized PCa patients with biopsy GS 9-10, who either received RP 土 aRT or EBRT, from within the SEER database.	Propensity score matching was followed by the use of multivariable competing-risks regression analysis, cumulative incidence plots, and temporal trends to evaluate the impact of local therapy on CSM and OCM. Sensitivity analyses based on the primary treatment type (RP alone vs. EBRT) were carried out.	No CSM differences were observed after RP 土 RT vs. EBRT. However, in patients in whom RP did not have to be combined with a RT, RP seems to be associated with a minor improvement in cancer-specific survival compared to EBRT.
17	Barry W. Goy, 2019 [33]	USA	Retrospective cohort study	Intermediate-risk prostate cancer (IRPC) patients who were clinically staged by DRE, PSA and biopsy tests, and underwent RP, EBRT, or BT between January 2004 and December 2007.	The RP was open versus conventional laparoscopy. Using a 6-field method, EBRT patients received 3-dimensional conformal therapy with a planned target volume of 0.8 cm around the prostate and seminal vesicles, but 0.6 cm posterior. Over the course of eight weeks, the median dose to the isocenter was 75.3 Gray (range 73.5-77.1). Using fluoroscopy, stepper-stabilizer units, and ultrasound guidance, loose or stranded Iodine-125 radioactive seeds were implanted transperineally for BT. With a modified peripheral loading technique, 0.4 millicuries per seed were used to prescribe a minimum peripheral dose of 145 Gray.	BT using Iodine-125, used alone or in combination with supplemental external radiation, is a reasonable treatment option for IRPC patients, to yield equivalent rates of DMFS and PCSS.
18	Hideyasu Tsumura et al., 2022 [34]	Japan	Retrospective cohort study	Patients with intermediate-risk PCa who underwent SEED-BT 土 EBRT and RP at three tertiary hospitals between January 2006 and December 2011.	Either laparoscopic surgery or the open retropubic technique was used to perform RP. The therapy protocol of each institution dictated the prescribed dose of SEED-BT alone for patients, which was either 145 or 160 Gy. We utilized iodine-125 for every patient. The TG43 criteria were used to define the doses.	The BCR-free rates did not differ significantly between patients treated with SEED-BT and those treated with RP for intermediate-risk PCa, even when they were directly compared using the surgical definition for BCR.
19	Chad A. Reichard et al., 2019 [35]	USA	Retrospective cohort study	Men diagnosed with high-risk (biopsy Gleason sum 8–10 or serum PSA level >20 ng/ mL or clinical stage ≥T3) and very-high-risk (VHR; primary Gleason pattern 5 on biopsy or ≥5 biopsy cores containing Gleason sum 8–10 or multiple individual NCCN high-risk features) PCa who chose RP with pelvic lymphadenectomy or RT+ADT from 2004 to 2013; in a MDPCC.	The analysis of outcome differences between treatment groups was conducted using the log-rank test. The study employed multivariable Cox modeling to compare the effects of different therapy types on OS and both distant and local recurrence-free survival.	There was no difference in local recurrence, distant metastasis failure and OS between patients undergoing RP vs RT+ADT. Patients treated via the MDPCC survived on average 16.9 months longer than those in the matched SEER cohort.

CASP Assessment

The quality assessment of included studies was conducted using the CASP tool, as summarized in Table 2.

**Table 2:** Quality Assessment using Critical Appraisal Skill Program (CASP) tool Y: yes; N: no; ?: cant tell; DMFS: distant metastasis free survival; PCSS: prostate cancer-specific survival; OS: overall survival; RP: radical prostatectomy; EBRT: external beam radiation therapy; BT: brachytherapy' CI: confidence interval; HR: adjusted hazards ratio

Sr. #	Questions	Benedikt Hoeh, 2021 [17]	Ugo Giovanni Falagario, 2023 [18]	Nikhil T. Sebastian, 2019 [19]	Kirsti Aas, 2021 [20]	Derya Tilki, 2018 [21]	Francesco Chierigo & Marco Borghesi, 2022 [22]	Fei Wang, 2021 [24]	Seok-Joo Chun, 2021 [26]	N. Sanmamed, 2023 [28]	Ahmed Emam, 2021 [30]	Barry W. Goy, 2019 [33]	Hideyasu Tsumura et al., 2022 [34]
1	Was the study addressing a clearly defined problem?	Y	Y	Y	?	Y	Y	Y	Y	Y	Y	Y	Y
2	Did the authors address their research questions appropriately?	Y	Y	Y	N	N	Y	Y	Y	N	Y	Y	Y
3	Did the cases get recruited appropriately?	?	?	Y	Y	Y	?	?	?	?	Y	?	?
4	Were the controls chosen in a way that made logical sense?	?	Y	Y	N	Y	Y	?	?	Y	Y	?	Y
5	Was the bias minimized by accurately measuring the exposure?	Y	Y	?	Y	?	Y	Y	Y	Y	Y	Y	?
6(a)	Were the groups given the same treatment aside from the experimental intervention?	Y	Y	Y	?	?	?	Y	Y	?	Y	?	Y
6(b)	Have the authors considered any potential confounding variables in their analysis or design?	N	N	?	N	N	?	?	Y	?	?	?	?
7	How large was the treatment’s effect?	HR=0.42 with 95% CI=0.20-0.89	HR=0.66 with 95% CI=0.57-0.75	HR=1.24 with 95% CI=0.58-2.65	HR=0.49 with 95% CI=0.24-0.96	HR=1.33 with 95% CI=0.49-3.64	HR=0.66 with 95% CI=0.52-0.86	HR=1.09 with 95% CI=1.02-1.16	No significant difference in DMFS, PCSS, or OS was found in the propensity score matching analysis	No statistical difference was found in PCSS	No statistical difference	No statistical difference	HR=8.072 with 95% CI=0.159-408.3
8	To what extent was the treatment effect estimate accurate?	Statistically significant association with p-value=0.02	p-value not computed	No statistically significant difference in OS between RP and EBRT+BT	Statistically significant association with p-value 0.039	P=0.58, not statistically significant	Statistically significant association with p<0.001	P=0.0069; the statistically significant association was found	p=0.068	P-value not computed	P-value not computed	P-value not computed	P=0.296, not statistically significant
9	Do you think that the outcomes are credible?	?	Y	?	N	?	Y	Y	?	Y	Y	?	Y
10	Can the local population use these results?	Y	?	Y	?	Y	Y	Y	Y	Y	Y	Y	Y
11	Are the findings of this study consistent with other available data?	Y	Y	Y	N	N	Y	Y	Y	Y	Y	N	N
	SCORE OUT OF 11	6	7	7	4	4	7	7	6	6	9	4	5

Forest Plots

Seven forest plots were created using data from thirteen distinct studies, with the data primarily being reported as the generalized inverse variance measured by the hazard ratio (HR). To compute the HR in terms of "log[HR]" and standard error "(SE)," a random-effects model was selected. The horizontal axis was used to calculate the confidence interval (CI=95%), and the plot's "point estimation" was presented as red squares. The vertical line in the center denotes a condition of "no effect."

Prostate Cancer-Specific Mortality

**Figure 2:**
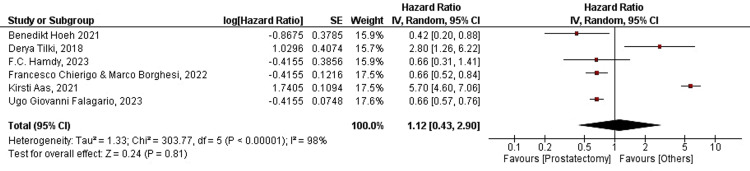
Forest plot for PCSM in RP-treated patients RP: radical prostatectomy; PCSM: prostate cancer-specific mortality; CI: confidence interval [17,18,20,21,22,27]

For radical prostatectomy: A forest plot for six out of 19 studies was plotted for PCSM in patients treated with RP. Four out of six (66.6%) studies favored prostatectomy, while two out of six (33.3%) advocated for interventions other than prostatectomy. The overall HR was found to be HR=1.12, CI=95% (0.43-2.90). Heterogeneity was found to be Tau²=1.33, Chi²=303.77, df=5, and I²=98%. In the current analysis, the overall effect size was represented in terms of Z, where Z=0.24 (p-value=0.81). The overall analysis did not favor RP. We concluded that RP failed to significantly improve PCSM. Interestingly, the individual effect sizes favored RP in terms of the cumulative incidence of cancer mortality in most studies. The overall HR was found to be HR=0.42, CI=95% (0.20-0.88) for Benedikt Hoeh (2021), whereas HR for Derya Tilki (2018) was found to be HR=2.80, CI=95% (1.26-6.22). Further, the HRs were HR=0.66, CI=95% (0.31-1.41), HR=0.66, CI=95% (0.52-0.84), HR=5.70, CI=95% (4.60-7.06), and HR=0.66, CI=95% (0.57-0.76) for Freddie C. Hamdy (2023), Francesco Chierigo and Marco Borghesi (2022), Kirsti Aas (2021), and Ugo Giovanni Falagario (2023), respectively. The forest plot for PCSM in patients treated with RP is shown below in Figure 2.

**Figure 3:**
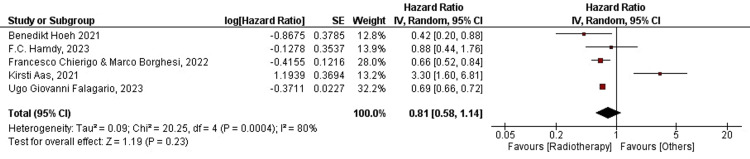
Forest plot for PCSM in RT-treated patients RT: radiation therapy; PCSM: prostate cancer-specific mortality; CI: confidence interval [12,17,18,20,27]

For radiation therapy: Forest plots for five of the 19 studies were plotted for PCSM in patients treated with RT. Data for the current intervention were extracted from the same studies to ensure an effective comparison between the interventions due to coherence in population demographics. One study was excluded because the data were incomparable and non-poolable. Four out of five (80%) studies favored RT, whereas one out of five (20%) advocated interventions other than RT. The overall HR was found to be HR=0.81, CI=95% (0.58-1.14). Heterogeneity was found to be Tau²=0.09, Chi²=20.25, df=4, and I²=80%. In the current analysis, the overall effect size was represented in terms of Z, where Z=1.19 (p-value=0.23). The overall analysis was found to favor RT, which supports the results of our previous analysis. We concluded that treatment with RT significantly improved PCSM. The average HR was found to be HR=0.42, CI=95% (0.20-0.88) for Benedikt Hoeh (2021), whereas the HR for Freddie C. Hamdy (2023), was found to be HR=0.88, CI=95% (0.44-1.76). Further the HRs were HR=0.66, CI=95% (0.52-0.84), HR=3.30, CI=95% (1.60-6.81), and HR=0.69, CI=95% (0.66-0.72) for Francesco Chierigo and Marco Borghesi (2022), Kirsti Aas (2021), and Ugo Giovanni Falagario (2023), respectively. The forest plot for PCSM in patients treated with RT is shown below in Figure 3.

Prostate Cancer-Specific Survival

**Figure 4:**
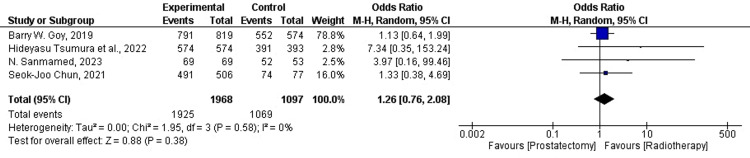
Forest plot for PCSS in RP and RT-treated patients RP: radical prostatectomy; PCSM: prostate cancer-specific mortality; CI: confidence interval; RT: radiation therapy [26,28,33,34]

One of the primary endpoints of the current analysis was prostate cancer-specific survival (PCSS) (five or 10 years) in patients treated with RP or RT. A forest plot for four of the 19 studies was plotted for dichotomous data. The sample size changed significantly between the treatment groups (N=1968 for RP and N=1097 for RT). Four out of four (100%) of the studies favor RT. The overall Mantel-Haenszel (M-H) OR was found to be M-H=1.26, CI=95% (0.76-2.08). Heterogeneity was found to be Tau²=0.00, Chi²=1.95, df=3, and I²=0%. In the current analysis, the overall effect size was represented in terms of Z, where Z=0.88 (p-value=0.38). The overall analysis was found to favor RT, which is in concordance with the results of our previous analyses. We conclude that RT treatment is associated with better PCSS. The M-H ratio was found to be M-H=1.13, CI=95% (0.64-1.99) for Barry W. Goy (2019); whereas the M-H ratio for Hideyasu Tsumura et al. (2022) was found to be M-H=7.34, CI=95% (0.35-153.24). Further, the M-H ratios were M-H=3.97, CI=95% (0.16-99.46), and M-H=1.33, CI=95% (0.38-4.69) for N. Sanmamed (2023), and Seok-Joo Chun (2021), respectively. This analysis conforms to our previous findings, which demonstrated that RT, not prostatectomy, significantly lowered the PCSM in patients with advanced-stage PCa. For the reported data, the M-H analysis provided a comparison between the treatment groups. It further showed whether the OR was equal (homogenous) or unequal (heterogeneous) across all populations. The forest plot for PCSS in patients treated with RP and RT is shown below in Figure 4.

Overall Mortality

**Figure 5:**
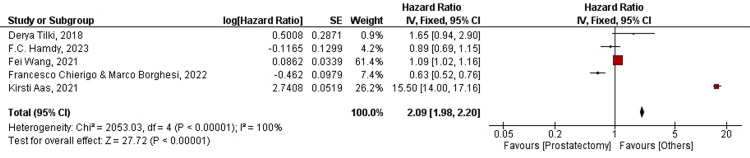
Forest plot for OM in RP-treated patients OM: overall mortality; RP: radical prostatectomy; CI: confidence interval [20,21,22,24,27]

For radical prostatectomy: Another primary endpoint of our analysis was ACM/OM in patients treated with RP or RT. A forest plot for five out of the 19 studies was plotted for OM in patients treated with RP. Two out of five (40%) studies favored prostatectomy, while three out of five (60%) advocated for interventions other than prostatectomy. The overall HR was found to be HR=2.09, CI=95% (1.98-2.20). Heterogeneity was found to be Chi²=2053.03, df=4, and I²=100%. In the current analysis, the overall effect size was represented in terms of Z, where Z=27.72 (p-value<0.00001). The overall analysis did not favor RP. We concluded that RP failed to significantly improve the OM. The average HR was found to be HR=1.65, CI=95% (0.94-2.90) for Derya Tilki (2018), whereas the hazard ratio for F.C. Hamdy (2023) was found to be HR=0.89, CI=95% (0.69-1.15). Further, the HRs were HR=1.09, CI=95% (1.02-1.16), HR=0.63, CI=95% (0.52-0.76), and HR=15.50, CI=95% (14.00-17.16) for Fei Wang (2021), Francesco Chierigo and Marco Borghesi (2022), and Kirsti Aas (2021), respectively. In the current analysis, we found a negative association between RP and OM. OM was found to be independent of the type of intervention conducted in patients with PCa. The forest plot of OM in patients treated with RP is shown below in Figure 5.

**Figure 6:**
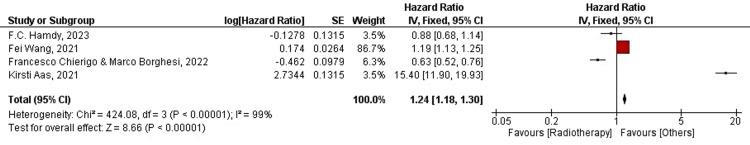
Forest plot for OM in RT-treated patients OM: overall mortality; RT: radiation therapy; CI: confidence interval [20,22,24,27]

For radiation therapy: Forest plots for four out of the 19 studies were plotted for OM in patients treated with RT. Data for the current intervention were extracted from the same studies to ensure an effective comparison between the interventions due to coherence in population demographics. One study was excluded because the data were incomparable and non-poolable. Two out of four (50%) studies favored RT, while two out of four (50%) advocated interventions other than RT. The overall HR was found to be HR=1.24, CI=95% (1.18-1.30). The heterogeneity was Chi²=424.08, df=3, and I²=99%. In the current analysis, the overall effect size was represented in terms of Z, where Z=8.66 (p-value<0.00001). The overall analysis did not favor RT. We concluded that there was a negative association between RT and OM. The average HR was found to be HR=0.88, CI=95% (0.68-1.14) for Freddie C. Hamdy (2023), whereas HR for Fei Wang (2021) was found to be HR=1.19, CI=95% (1.13-1.25). Furthermore, the HRs were HR=0.63, CI=95% (0.52-0.76), and HR=15.40, CI=95% (1.90-19.93) for Francesco Chierigo, Marco Borghesi (2022), and Kirsti Aas (2021), respectively. Similar to the previous analysis for OM, we concluded that the two variables, RT and OM, are independent, and their results do not impact each other. The forest plot for OM in patients treated with RT is shown below in Figure 6.

Furthermore, the comparative analysis of both interventions indicated that patients who underwent RT were at a greater risk for OM than those who underwent prostatectomy.

Overall Survival

**Figure 7:**
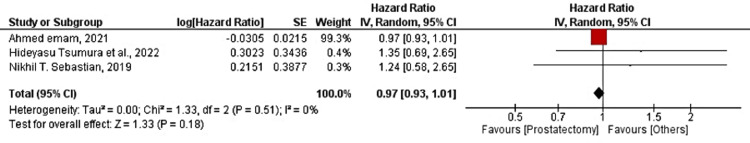
Forest plot for OS in RP-treated patients OS: overall survival; RP: radical prostatectomy; CI: confidence interval [19,30,34]

For radical prostatectomy: One of the primary endpoints of the current analysis was OS in patients treated with RP or RT. A forest plot for three of the 19 studies was plotted for OS in patients treated with RP. Only one out of three (33.3%) studies favored prostatectomy, while two out of three (66.6%) advocated interventions other than prostatectomy. The overall HR was found to be HR=0.97, CI=95% (0.93-1.01). Heterogeneity was found to be Tau²=0.00, Chi²=1.33, df=2, and I²=0%. In the current analysis, the overall effect size was represented in terms of Z, where Z=1.33 (p-value=0.18). The overall analysis was in favor of RP. We concluded that RP was associated with significantly improved OS. Interestingly, the individual effect sizes of the majority of studies favored interventions other than RP in terms of OS; however, one study had high accuracy and therefore occupied a larger weight in the analysis. As a result, our findings shifted in favor of RP. The average hazard ratios were HR=0.97, CI=95% (0.93-1.01), HR=1.35, CI=95% (0.69-2.65) and HR=1.24, CI=95% (0.58-2.65) for Ahmed Emam (2021), Hideyasu Tsumura et al. (2022) and Nikhil T. Sebastian (2019), respectively. The forest plot for OS in patients treated with RP is shown below in Figure 7.

**Figure 8:**
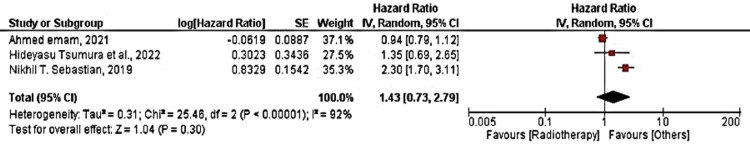
Forest plot for OS in RT-treated patients OS: overall survival; RT: radiation therapy; CI: confidence interval [19,30,34]

For radiation therapy: Forest plots for three of the 19 studies were plotted for OS in patients treated with RT. Data for the current intervention were extracted from the same studies to ensure an effective comparison between the interventions due to coherence in population demographics. One out of three (33.3%) studies favored RT, while two out of three (66.6%) advocated interventions other than RT. The overall HR was found to be HR=1.43, CI=95% (0.73-2.79). Heterogeneity was found to be Tau²=0.31, Chi²=25.46, df=2, and I²=92%. In the current analysis, the overall effect size was represented in terms of Z, where Z=1.04 (p-value=0.30). The overall analysis was not found to favor RT, which supports the results of our previous analysis. We concluded that RT was not associated with an improved OS. The average HRs were HR=0.94, CI=95% (0.79-1.12), HR=1.35, CI=95% (0.69-2.65), and HR=2.30, CI=95% (1.70-3.11) for Ahmed Emam (2021), Hideyasu Tsumura et al. (2022), and Nikhil T. Sebastian (2019), respectively. The forest plot for OS in patients treated with RT is shown below in Figure 8.

Discussion

For localized PCa, RP and EBRT are acknowledged as comparable treatment choices. Previous investigations comparing oncological outcomes following surgery and RT have shown the comparable nature or potential superiority of surgery. However, there is still disagreement regarding the superior course of treatment. Differing definitions of biochemical recurrence (BCR), disparate complication profiles across treatment modalities, and disparate patient backgrounds due to selection bias complicate a meaningful comparison of their results. Additionally, each treatment option has disadvantages regarding functional outcomes, such as side effects from the medication or issues with continence, potency, and QoL. This further complicates the process of selecting the optimal treatment. Hemorrhage, damage to surrounding organs (particularly the rectum), infection at the surgical site, hernias in the abdominal wall and inguinal canal, urinary incontinence, and erectile dysfunction are among the main side effects of RP. Radiation cystitis, diarrhea, dermatitis, rectal bleeding, vesical hemorrhage, and secondary malignancies are among the most common side effects of EBRT. The first large-scale RCT comparing EBRT, active monitoring, and RP for localized PCa was published in 2016 [5]. More recently, a different study compared the effectiveness of volumetric modulated arc therapy and robot-assisted RP, the two most popular RT and surgery approaches, respectively [36]. Additionally, BT boost in conjunction with EBRT is becoming an increasingly common treatment strategy for patients with high-risk PCa, and recent evidence has shown that it is associated with significantly lower rates of metastasis [37]. The current study compiled the most recent data on the oncological effects of surgery versus EBRT for locally advanced PCa.

In this systematic review and meta-analysis, we compared the outcomes of RP and RT with each other regarding PCSM and OM. Data on PCSS and OS were also analyzed in relevant studies for a comprehensive understanding and drawing of results. The results from our systematic review showed a clear superiority of RP over RT as the primary treatment for PCa. RP has the potential to greatly enhance survival rates compared with other available treatment methods. This is evident from the results of our systematic review, which revealed a total of 12 out of 19 (63%) studies advocating the effectiveness of RP for the treatment of PCa. In contrast, one out of 19 (5.3%) studies advocated RT or BT with or without ADT to be effective in PCa treatment. Six out of 19 (31.6%) studies also concluded that there was “no statistical difference” in either treatment modality in the outcomes of patients with PCa. The mortality rates became almost equal across the treatment groups in a lot of studies where EBRT was used in combination with BT [19,22]. However, RP was associated with an increased BCR compared to EBRT [18,25,30]. RT was not only associated with an increased mortality rate but also an increased risk of recurrence, metastases, biochemical failure, and clinical progression [18,24,27].

Contrary to the literature in our review, the results of our synthesis proved that PCSM is lower in patients treated with RT. The total effect size for the analysis was calculated as Z=1.19 (p-value=0.23). The heterogeneity in the studies was Tau2=0.09, Chi2=20.25, df=4, and I2=80% (Figure 3). Moreover, OS was shown to be higher in RP-treated patients (Figure 7; the analysis is highly accurate with I²=0%). The combined effect for the analysis was found to be: HR=0.97 (0.93, 1.01). The total effect was calculated as Z=1.33 (p-value=0.18). The heterogeneity was found to be Tau2=0.00, Chi2=1.33, df=2, and I2=0%. However, OM was shown to be independent of either treatment modality (Figures 5, 6). PCSS was shown to favor RT (Figure 4), with the analysis having very high accuracy (I²=0%). The p-value is less than 0.05 due to low-power cohort studies being included in the meta-analysis. RP and RT also had different QoL outcomes. Nonetheless, studies that comprehensively compare RP and RT with respect to functional results and health-related QoL are lacking. While erectile dysfunction and acute and late genitourinary or gastrointestinal toxicity are among the other side effects of RT, the risk of incontinence is lower with RT than with RP [38]. In one study, the incidence of complications following RP and RT was examined; 10% of patients required RP pads, but 0% after RT. Conversely, 10% of RT patients experienced grade ≥3 rectal bleeding or hematuria, compared to 0% following RP [39]. Thus, carefully planned prospective trials that compare the side effects and functional outcomes of the two therapy modalities are required.

Guidelines should be optimized based on such reviews and similar future research on this subject. Medical practitioners should recommend RP for high-risk PCa patients, considering the risk of increased BCR in patients with locally advanced disease and other risk factors such as age and their correlation with other patient outcomes. Depending on the illness risk and patient life expectancy, current recommendations in real-world clinical practice prescribe either RP or EBRT. For instance, the European Association of Urology (EAU)-European Society for Radiotherapy & Oncology (ESTRO)- International Society of Geriatric Oncology (SIOG) Guidelines on PCa do not strongly recommend active treatment (RP or EBRT) for low-risk patients, but they recommend RP for intermediate- or high-risk patients with a life expectancy of >10 years and EBRT in combination with ADT for intermediate- or high-risk patients, respectively [40]. Similarly, the top age limit for surgery is not specified by the current clinical guidelines, even though they often advise RP for patients with a life expectancy >10 years [41]. In actual clinical settings, doctors should inform patients about their complication profiles and consider their preferences before determining the best course of action. Our results support some of the findings of previous reviews and meta-analyses on this topic. For instance, in a study conducted by Abdulmajeed Aydh in 2021, EBRT alone had a significantly worse OS than RP [42]. The results were also in agreement with a study conducted in 2017 by Omar Fahmy, who also reported a significant improvement in OS in RP-treated patients [43].

Strengths

The search strategy utilized in our study ensured the inclusion of a range of studies, providing a broad representation of the current literature on RT versus surgical intervention for the treatment of PCa. Rigorous inclusion criteria were applied to strengthen the quality of the research and minimize the risk of bias. Additionally, the use of a meta-analytic approach allowed for the quantitative synthesis of data, enabling the assessment of the associated efficacy and long-term patient outcomes. These methodological strengths collectively contribute to the robustness of the study's conclusions and offer valuable insights into optimal treatment strategies for PCa.

Limitations

Although the study investigated the right outcomes and measures for analysis and assessment, it had several limitations owing to limitations in the constituent studies. First, a comparative analysis of both treatment modalities was not always performed in every study. Second, the sample sizes were highly variable, ranging from as small as 156 to as large as 137,741, and the available data required frequent standardization. Often, the sample sizes taken for meta-analysis could not be standardized according to usual protocols. Moreover, population demographics were not homogenous across all studies because studies from all over the globe were included. Most of the studies available were of the cohort type, which decreased the reliability of our results. Third, very few primary studies were utilized to assess the effectiveness (outcome domain) of such a large sample size. Fourth, we evaluated the overall combined effect of all sample sizes; however, within-group and subgroup analyses were not performed. Several studies have demonstrated that the results of the final analysis can be significantly altered when population demographics are subgrouped into effect sizes. Finally, moderator analysis was not performed because of incoherent and incomparable data.

## Conclusions

In light of the findings from our systematic review and meta-analysis comparing RP and RT for localized PCa, our results demonstrate that PCSM tends to be lower in patients treated with RT, while OS appears to be higher in RP-treated patients. Importantly, OM was found to be independent of the type of intervention. These results highlight the complexity of treatment decisions in PCa management and emphasize the need for further research to guide medical practitioners in recommending optimal treatment strategies tailored to individual patient characteristics and preferences, with the ultimate goal of achieving improved patient outcomes.
